# Shear Bond Strength of Rebonded Ceramic Brackets Using Four Different Methods of Adhesive Removal

**Published:** 2018-01

**Authors:** Amir Hossein Mirhashemi, Mohammad Hashem Hosseini, Nasim Chiniforoush, Armin Soudi, Meisam Moradi

**Affiliations:** 1 Associate Professor, Dental Research Center, Dentistry Research Institute, Tehran University of Medical Sciences, Tehran, Iran; Department of Orthodontics, School of Dentistry, Tehran University of Medical Sciences, Tehran, Iran; 2 Assistant Professor, Dental Research Center, Dentistry Research Institute, Tehran University of Medical Sciences, Tehran, Iran; Department of Orthodontics, School of Dentistry, Tehran University of Medical Sciences, Tehran, Iran; 3 Researcher, PhD of Laser Dentistry, Laser Research Center of Dentistry, Dentistry Research Institute, Tehran University of Medical Sciences, Tehran, Iran; 4 Postgraduate Student, Department of Orthodontics, School of Dentistry, Tehran University of Medical Sciences, Tehran, Iran

**Keywords:** Lasers, Recycling, Orthodontic Brackets

## Abstract

**Objectives:**

Rebonding of isolated brackets is an economic option that can be conducted using available in-office or commercial recycling methods. Nowadays, lasers are known as an efficient modality for composite removal, but there is not much information available about using lasers for removal of adhesive remnants from the ceramic bracket base.

**Materials and Methods:**

Fifty human premolar teeth were divided into five groups. Samples in all groups were bonded to ceramic brackets. Brackets in four groups were debonded and the remaining adhesive was removed by Er:YAG laser, Er;Cr:YSGG laser, sandblasting or direct flame. After removing adhesives from the tooth surfaces by carbide bur, the recycled brackets were bonded again. in the control group, new ceramic brackets were bonded. Finally, all brackets were debonded by universal testing machine and their shear bond strength (SBS) was measured. The adhesive remnant index (ARI) was calculated under a stereomicroscope at ×10 magnification. Data were analyzed using oneway ANOVA and Tukey’s test.

**Results:**

SRS values showed no significant difference among the five groups (P=0.568). The highest SRS was noted in the control group (7.46±1.4 MPa), followed by Er:YAG laser group (7.40±1.24 Mpa) and the lowest was noted in the flame group (6.32±2.3 Mpa). ARI scores indicated that most of the adhesive remained on the tooth surface in all groups

**Conclusions:**

Recycling of ceramic brackets with Er:YAG laser is an efficient in-office method which causes the least damage to the bracket base. However, all methods of bracket recycling showed acceptable SBS.

## INTRODUCTION

Fixed orthodontic treatment requires an effective bonding between brackets and enamel surfaces [1]. These appliances include attachments that are bonded directly to the tooth and should remain in place until the end of treatment [2]. The bond between bracket base and enamel surfaces should be strong enough to resist shear forces and tensions [3]. One expected problem in orthodontic treatment is bond failure between the tooth surface and bracket, with a prevalence of about 17.6% [4]. Bracket debonding is not unusual during orthodontic treatment and is mainly due to bite forces and low bond strength. In addition, improper placement of bracket may necessitate bracket repositioning [5,6]. There is a tendency to simplify technical methods in orthodontics to reduce treatment costs, like other fields of dentistry [2].

Thus, bracket rebonding is considered as a cost-effective option and it has considerable advantages for clinical work [4,7]. It seems logical to recycle brackets instead of using new ones, which can lead to decrease costs. The main purpose of the recycling process is to remove adhesives from the bracket base without damaging it or changing the bracket slot dimensions [3,4,7–9]. The shear bond strength (SBS) of recycled brackets is affected by several factors including microscopic destruction of bracket base, bracket base design and the adhesive remnants on the base and also method of bracket removal [4,10].

Ceramic brackets are bonded to tooth surfaces via three different mechanisms: mechanical bonding, chemical bonding or a combination of both. Considering enamel fractures during debonding of ceramic brackets with chemical retention, a new generation of ceramic brackets with mechanical retention was invented [11]. The bond strength of ceramic brackets with mechanical retention was found to be equal to or less than that of stainless steel brackets [12]. The rate of failure for ceramic brackets with mechanical retention has not been reported. Ceramic brackets are certainly more fragile than the commonly used metal brackets. Thus, they are probably prone to fracture during debonding. However, debonded brackets which remain intact do not lose their angulation accuracy, torque and contour of the base [6,9,13]. Based on this information, recycling of debonded brackets instead of using new brackets is cost-saving. However, the base of ceramic brackets is fragile and more prone to damage compared to metal brackets. Thus, selecting an appropriate in-office method to prepare ceramic brackets is challenging [11].

Different methods have been suggested to remove the remaining adhesive from the ceramic bracket base for rebonding [11]. In-office methods such as heating methods (direct heating), mechanical methods (sandblasting or using green stone or tungsten carbide bur) [6,14,15] and recently laser have been used for bracket recycling. Demand for use of laser in dentistry has increased during the past years [1,4]. Lasers such as Er:YAG, Nd:YAG, Er,Cr:YSGG and CO2 are used for removal of adhesive remnants [1]. Development of Er:YAG laser and recently Er,Cr:YSGG enabled the removal of composite from the bracket base or tooth surfaces completely with no destructive side effect [5].

Most previous studies have focused on rebonding of ceramic brackets with chemical retention and studies on rebonding of ceramic brackets with mechanical retention are limited. This study aimed to find an appropriate method to remove adhesives from the debonded ceramic brackets.

## MATERIALS AND METHODS

Fifty maxillary first premolars extracted for orthodontic purposes with no caries, cracks or restorations were cleaned from soft tissue residues and immersed in 0.5% chloramine T solution at 4°C for seven days. After that, the samples were transferred to distilled water until performing the study. Based on a previous study [16], type of storage media and storage time have no or little effect on bond strength. The study was approved in the ethics committee of our university (ethical approval code:878).

The teeth were cleaned from the soft tissue remnants and they were examined by a stereomicroscope at ×10 magnification to ensure they had no obvious enamel cracks. Teeth with cracks in their buccal surface were excluded from the study. In the next step, samples were polished with a rubber cup and pumice powder for 15 seconds to remove debris from their surface.

### Sample preparation and group allocation:

Forty new ceramic brackets were bonded to wet non-etched tooth surfaces with Transbond XT adhesive to facilitate debonding [17]. The brackets which were distorted during debonding were replaced with new brackets. Ten teeth were used as controls to assess SBS of ceramic brackets.

The debonded ceramic brackets were divided into four groups:

1. In the flaming group, debonded ceramic brackets were subjected to burning process which consists of these steps: heating the bracket bases by a torch (Honset 500 Jet, Beijing, China), burning the adhesive, washing the base with high water vapor pressure, cleaning the base by ultrasonic bath for five minutes and complete drying.
2. In the sandblasted group, brackets were cleaned using 50 μm aluminum oxide abrasive particles (Venfert, Ibbenbüren, Germany) with 10 mm distance between the bracket base and hand piece head until no adhesive remnant can be detected by the naked eye. The remaining powder was cleaned from the bracket base by air spray for 15 seconds.
3. In the Er:YAG (Doctor Smile, Brendola, Italy) group, brackets were irradiated with Er:YAG laser at a wavelength of 2940 nm (4W output power, 10 Hz frequency, 600μm tip diameter, 90% air output and 70% water output) and 6 mm distance. During laser irradiation the bracket base was perpendicular to the laser to remove adhesive. Operators wore protective glasses during laser irradiation.
4. In the Er,Cr:YSGG (Biolase, water lase, Irvine, USA) group, the brackets were irradiated with laser at a wavelength of 2780 nm (4W output power, 20 Hz frequency, 600μm tip diameter, 90% air output and 70% water output) and at a distance of 6 mm. During laser irradiation, the bracket base was perpendicular to the laser to remove adhesive. Operators wore protective glasses during laser irradiation.

After preparing the samples, debonded ceramic brackets were bonded in the control group as follows:

For etching of the buccal surface of the teeth, 32% phosphoric acid (Scotchbond Universal etchant; 3M ESPE, St. Paul, MN, USA) was used according to the manufacturer’s instructions. The teeth were etched for 15 seconds, rinsed and dried to obtain a chalky white appearance. We used primer (Transbond XT) for bonding of brackets. Transbond XT composite (3M Unitek, Monrovia, CA, USA) was applied on standard ceramic edgewise premolar bracket base (GAC, USA) with 0.018-inch slot size according to the manufacturer’s instructions.

Brackets were then bonded to the middle part of the buccal surface by applying equal force. After aligning the longitudinal axis of the bracket parallel to the longitudinal axis of the tooth, excess adhesive was removed by a scaler and light curing was performed using a light curing unit (Woodpecker, London, UK) with 1200 mW/cm^2^ intensity. Each tooth was cured for 40 seconds from the mesial, distal, occlusal and gingival surfaces. All procedures were performed by the same operator. The samples were immersed in distilled water at 37°C for 24 hours. The samples were then subjected to 1500 thermal cycles between 5–55°C with a dwell time of 20 seconds and transfer time of 10 seconds. The teeth were then mounted in molds measuring 2.5 cm. The internal surface of the mold was coated with petroleum jelly and the teeth were fixed using 16×22 inch rectangular stainless steel ligature wire. Each tooth was positioned at the center of the mold and the rectangular wire was fixed to the mold using sticky wax so that the teeth remained fixed when applying acrylic resin. Auto-polymerizing acrylic resin was applied to the mold and the teeth were embedded in acrylic to the level of their cementoenamel junction. After polymerization of acrylic resin, the teeth in acrylic blocks were separated from the mold.

### SBS testing:

Universal testing machine (Zwick Roell, Ulm, Germany) was used for SBS testing. The teeth were placed in the machine such that the bracket base was parallel to the load application vector. Load was applied in occlusogingival direction at a crosshead speed of 0.5 mm/minute to the bracket-tooth interface. Load at debonding was recorded in Newtons (N) and converted to Megapascals (MPa) by dividing the load in Newtons by the bracket base surface area in square-millimeters (mm^2^).

### Adhesive remnant index (ARI) scores:

The remaining adhesive on the tooth surface was observed under a stereomicroscope at ×10 magnification. Classification of ARI score was based on the study by Oilver and Pal [18].

1. All adhesive is left on the tooth enamel.
2. 90% of adhesive is left on the tooth enamel.
3. 10–90% of adhesive is left on the tooth enamel.
4. Less than 10% of adhesive is left on the tooth enamel.
5. No adhesive is left on the tooth enamel.

To evaluate the SBS, one-way ANOVA and to compare the different groups, Tukey’s test was used.

## RESULTS

This study was done to compare the SBS of ceramic brackets in five groups. Among the groups, maximum SBS was noted in the control group followed by Er:YAG laser group (7.4 Mpa) and the minimum SBS was noted in direct flame group (6.33 Mpa). Although Er:YAG laser group had the maximum SBS among the recovered groups, this difference was not statistically significant (P=0.568, Table 1).

**Table 1.** Shear bond strength of reboded ceramic brackets to the teeth in different adhesive removal groups (n=10)

**Group name**	**Minimum**	**Maximum**	**Mean**	**Standard deviation**
Control	5.77	9.54	7.46	1.40
Er:YAG	5.21	9.99	7.40	1.24
Er,Cr:YSGG	5.08	8.55	6.48	1.24
Flame	2.68	10.47	6.32	2.30
Sandblasting	3.53	11.75	6.83	2.84

### ARI:

The ARI values of the groups are shown in Table 2.

**Table 2.** Distribution of adhesive remnant index (ARI) scores in different surface treatments

**ARI Groups**	**1**	**2**	**3**	**4**	**5**
Control	10	0	0	0	0
Er:YAG	10	0	0	0	0
Er,Cr:YSGG	9	1	0	0	0
Sandblasting	9	1	0	0	0
Flame	5	3	2	0	0
Total	43	5	2	0	0

## DISCUSSION

Several techniques are used for recycling of orthodontic brackets to remove the remaining adhesives. These methods include air abrasion, wear by silicon carbide bur, microetching, lasers and industrial recycling procedures. Each method should provide acceptable bond strength, create less destructive side effects, be easy to use and less time consuming. The purpose of recycling is to remove the remaining adhesives completely from the bracket base without causing any damage or change the bracket slot dimensions. Although the required bond strength for clinical work has not been determined specifically and previous studies have reported different values, this parameter should be high enough for the bonded bracket to resist chewing forces. On the other hand, the bond strength should allow easy debonding of the bonded brackets without damaging the tooth enamel [19]. Matasa [20] stated that a bracket can be used for up to five times. Considering the increasing popularity and clinical use of ceramic brackets, there is a need for an effective way to recycle the brackets. Thus, this study was conducted to evaluate and compare the SBS of ceramic brackets with mechanical retention recovered by different methods. The SBS of new and recycled brackets is an interesting topic in orthodontic research.

Removal of the remaining resin and reuse of debonded brackets are less costly than the use of new brackets. In this study, the mean SBS of brackets in the control group was 7.46 Mpa. No significant difference was found between the groups, which is consistent with the results of Ishida et al [10]. In the study by Reynolds and von Fraunhofer [21] the SBS of 5.9 to 7.8 Mpa was introduced as the minimum required values for clinical practice. However, Mizrahi and Smith [22] concluded that bond strength in the range of 2.8 to 10 Mpa is sufficient for clinical purposes. The present study indicated that the bond strength of all groups was higher than the minimum range due to the anatomical diversity of the buccal surfaces of the teeth. This range can be affected by accurate placement of the machined blade. Most studies reported a wide range of diversity for bond strength [12,23,24].

Some researchers [25–27] reported higher values for the second bond strength while some others [28–32] reported higher values for the initial bond strength; such differences can be due to reasons including the type of studied tooth, using a new tooth for rebonding, the technique used for composite removal from the brackets or tooth surfaces, the bonding system, type of composite resin and inability to eliminate these confounders [1]. In the recent years, use of lasers in dentistry has increased. Preparation of metal brackets for rebonding by laser has been previously attempted with optimal results. However, complementary studies are required on ceramic brackets [33].

Er:YAG laser removes composite resin from tooth surfaces considerably more than ceramic materials. Thus, it is believed that adhesive removal from the ceramic bracket base is selective. This selective removal prevents excessive increase of temperature in ceramic brackets by use of air spray and water during the procedure [11].

In the present study, the maximum SBS of recycled ceramic brackets was found in this group (7.4 Mpa). Although this difference was not statistically significant in comparison to other groups, the results of this study indicated minimum reduction in bond strength of recycled brackets by Er:YAG laser. It can be concluded that the best method for recycling of ceramic brackets is to use Er:YAG laser. Han et al, [33] in their study concluded that irradiation of Er:YAG laser completely removes adhesive from ceramic bracket base without causing any damage. The SBS of ceramic brackets was similar to that of new brackets, which is consistent with our study results. In the study by Chacko et al, [5] on metal brackets, maximum amount of SBS was noted in Er:YAG laser group, similar to our study. However, in contrast to our study, the SBS of Er:YAG laser group had a significant difference with that of other groups.

In the study by Yassaei et al, [6] the SBS of recycled brackets by Er:YAG laser was about 13.40 Mpa which is almost twice the value in our results. The reason is probably the type of bracket and the laser settings. In their study, laser with 5.5 W power was used which was different from the laser power in our study. Another difference of their study with ours was that the values of the sandblasted group were higher than the values of laser irradiated group which can be attributed to the difference between the materials and size of the particles used.

Similar to Er:YAG laser, Er,Cr:YSGG laser works selectively and does not cause damage to the base unlike the sandblasting method [11]. In the present study, the SBS of this group was 6.48 Mpa which can be due to some degrees of damage to the bracket base. Ahrari et al, [11] in 2013 concluded that this laser has the ability to recycle debonded ceramic brackets with some degrees of damage to the bracket base with bond strength comparable to that of new brackets. In the study conducted by Ahrari et al, [11] to investigate the efficacy of this laser for composite removal, similar to our study, no significant difference was found between the bond strength of this laser compared to other groups. Although these lasers may be effective for composite removal, they are not used in common clinical work. They may even cause damage to the teeth. Previous authors showed the ability of this laser for removing restorative materials and roughening the surface of old composite restorations [34–36].

Sandblasting is commonly used to remove adhesives from the bracket base which is an effective method to recycle metal brackets [33]. In our study, the sandblasted group had a SBS about 6.83 Mpa which was the highest after the Er:YAG laser group. However, this difference was not statistically significant. These observations indicate that sandblasting can increase the SBS to the same level of the initial bond strength. These results were consistent with those of Basudan and Al-Emran [37]. The results of different studies on SBS of sandblasted groups can be diverse due to the difference in size of aluminum oxide particles and the duration of sandblasting. In addition, it can be due to the morphological changes in the bracket base which were confirmed by Millett et al, [38] and Arici et al [39]. In the study conducted by Han et al, [33] the SBS value of the sandblasted group was the lowest among the four groups of flaming, sandblasting, Er:YAG laser and control; this difference was statistically significant and therefore, inconsistent with our study results. In the study by Yassaei et al, [6] on the comparison of recycling of metal brackets by CO2 laser, Er:YAG laser, sandblasting and direct flame, sandblasting yielded the maximum amount of SBS, which was inconsistent with our study results. However, they obtained no significant difference between Er:YAG laser group and sandblasting group, which was in agreement with our results.

Yim et al. [40] compared five methods of heating, grinding with green stone and sandblasting of recycling ceramic and metal brackets for 4 and 8 seconds and concluded that sandblasting for 8 seconds caused maximum amount of SBS for metal brackets while the bond strength of ceramic brackets was less than that of the control group. The reason can be due to changes in metal bracket base and increased available surface for bonding.

Unlike most previous studies which reported high SBS with slight difference with the control group, in a study by Chung et al, [12] sandblasted recycled brackets showed almost one-fifth of the bond strength of new brackets. In another study by Geraldo-Martins et al, [41] to compare the effect of sandblasting and silica coating, it was found that the SBS of sandblasted brackets was less than that of other two groups (control and silica). In a study by Tavares et al, [3] no significant difference was found between the brackets in the control group, brackets recycled by sandblasting method and new brackets which were bonded to previously debonded teeth. However, they announced that the efficacy of the sandblasting depended on the type of bracket.

This method has been the most time-saving method compared to others [3,4]. This process needs adequate ventilation. Most studies on the efficacy of sandblasting for recycling of debonded metal brackets concluded that sandblasting increases micro-roughening and the available surface for bonding and therefore it increases mechanical retention [3,6].

Flaming is an old method to remove adhesives from the ceramic bracket base. Lew et al. [42] used this method for adhesive removal from the bracket base. They reported that bond strength of these processed ceramic brackets was significantly lower than that of other methods of bracket recycling. Their results were consistent with the results of our study. In our study, the mean SBS of the flaming group was minimum among all groups (6.32 Mpa). This finding was similar to the results obtained by Yassaei et al, [4] and Han et al [33]. Martina et al. [43] found that the SBS of brackets decreased when they were recycled by flaming and ultrasonic device. One of the problems of recycling of metal brackets with flaming is that these brackets may become cold-welded due to heat which decreases their resistance to corrosion [5].

Ceramic brackets are the only type of brackets that can maintain their dimensions during flaming. When ceramic brackets are heated, they show high resistance and maintain their slot form and dimensions after recycling [9]. However, When the composite resin is cracked and displaced from the ceramic bracket, it partly eliminates the irregularities in the zirconium layer at the base of the bracket which are created the manufacturers to increase bond strength by providing mechanical retention for the composite resin. This is in agreement with previous findings [44].

The ARI is an appropriate indicator for determining the amount of damage to the tooth structure during the debonding procedure. The results of ARI score assessment in our study indicated that most samples (n=43) had ARI score 1 which indicated that mechanical retention of brackets led to more adhesive remaining on the tooth enamel. Yassaei et al. [6] showed the same results. In a study by Ahrari et al, [11] brackets which were recycled by laser showed higher amount of adhesive remaining on the enamel.

There is a belief that rebonding of ceramic or non-damaged metal brackets is cost-saving. On the other hand, clinicians should pay attention to the time spent to clean and prepare the base of brackets for rebonding and the expenditure of additional materials or equipment for these techniques.

## CONCLUSION

Ceramic brackets recycled by Er:YAG laser had higher values of SBS than other groups close to that of the control group. Thus, Er:YAG laser may be used as an efficient method for recycling of these brackets in office. But in terms of clinical use, flame is more accessible and less costly to recycle ceramic brackets.
